# An update on the development of novel antifungal agents for eumycetoma

**DOI:** 10.3389/fphar.2023.1165273

**Published:** 2023-05-18

**Authors:** David J. Chandler, Alexandro Bonifaz, Wendy W. J. van de Sande

**Affiliations:** ^1^ Department of Global Health and Infection, Brighton and Sussex Medical School, Brighton, United Kingdom; ^2^ Dermatology Department, Brighton General Hospital, University Hospitals Sussex NHS Foundation Trust, Brighton, United Kingdom; ^3^ Hospital General de México “Dr. Eduardo Liceaga”, Mexico City, Mexico; ^4^ Erasmus MC, University Medical Center Rotterdam, Department of Medical Microbiology and Infectious Diseases, Rotterdam, Netherlands

**Keywords:** eumycetoma, Neglected Tropical Disease (NTD), mycology, skin NTDs, *Madurella mycetomatis*

## Abstract

Eumycetoma, a chronic subcutaneous mycosis, responds poorly to the available antifungal treatments and patients often require extensive surgical resection or amputation of the affected limb. More effective treatments are needed for eumycetoma. This article will describe some of the approaches being used to develop and evaluate new treatments for eumycetoma, summarise the latest developments and discuss the challenges that lie ahead.

## 1 Mycetoma—a Neglected Tropical Disease

**FIGURE 1:**
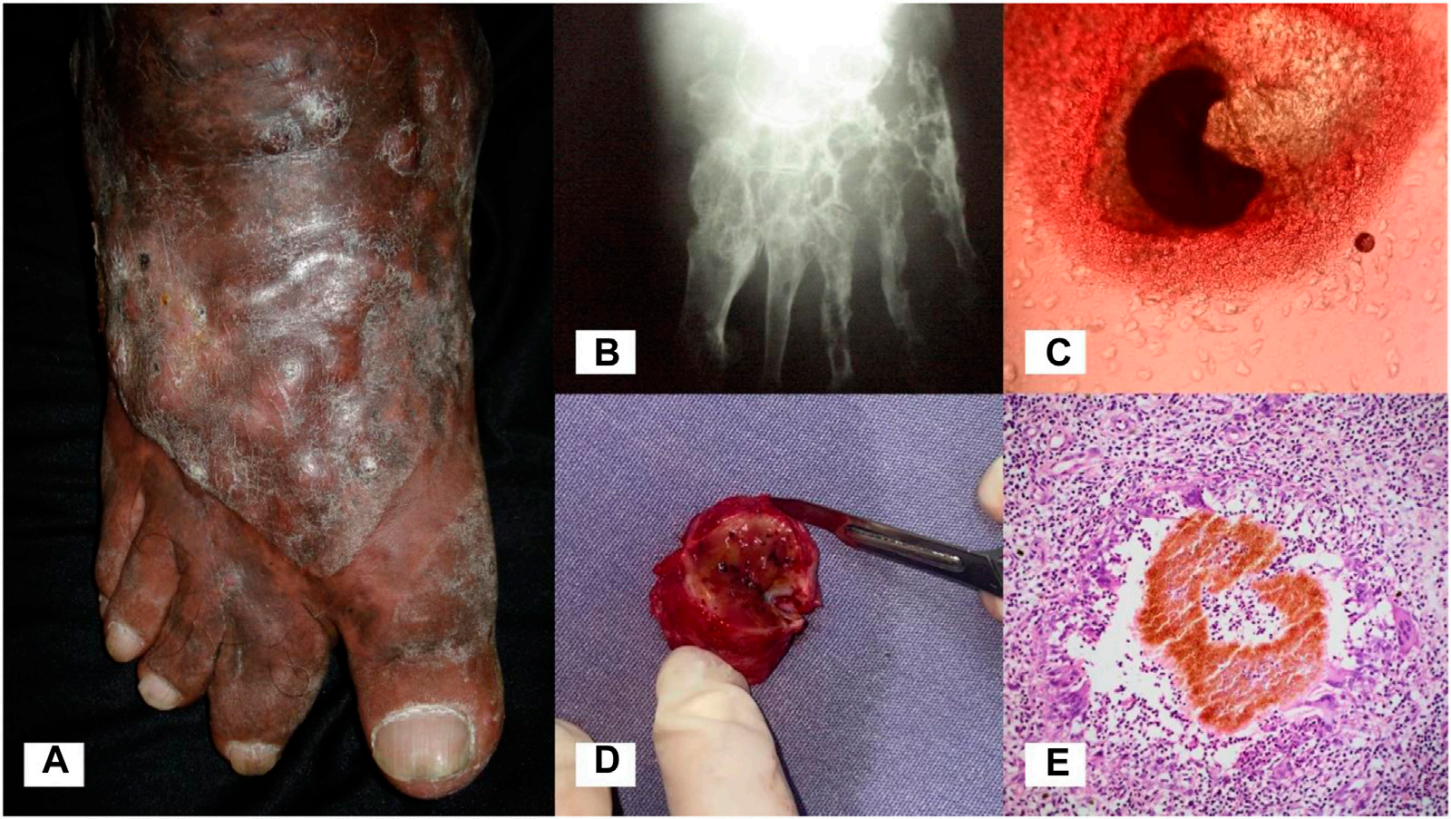
Eumycetoma. **(A)** Eumycetoma of the foot. **(B)** X-ray of the foot showing significant osteolysis and cavitating bone lesions. **(C)** Black grain of *Madurella pseudomycetomatis* on direct examination (KOH, 10x). **(D)** Encapsulated mycetoma, surgical specimen. Black grains in tissue are visible to the naked eye. **(E)** Skin biopsy showing a pigmented grain of *Madurella mycetomatis* (H&E, 10x).

Mycetoma is a chronic subcutaneous infection caused by aerobic actinomycetes (actinomycetoma) or fungi (eumycetoma) (Zijlstra et al., 2016). The causative agents of mycetoma exist in the environment and infection occurs following traumatic inoculation of the organism through the skin and into the subcutaneous tissue. Infection results in progressive swelling of the affected body part, most commonly the foot, leading to deformity and disability (Figures 1A,B). Characteristic of mycetoma is the formation of grains (compact microcolonies of the causative organism) in infected tissue (Figures 1C–E). Sinus tracts are formed within the subcutaneous mass, from which grains and purulent material are discharged.

Mycetoma, and the other subcutaneous fungal infections including sporotrichosis and chromoblastomycosis, are recognised by the WHO as Neglected Tropical Diseases (NTDs) (World Health Organization, 2016; World Health Organization, 2017; World Health Organization, 2020). The geographic distribution of mycetoma is not well understood. Most cases occur in low and middle income countries (LMICs) in tropical and subtropical regions between latitudes 30°N and 15°S, referred to as the “mycetoma belt”. Many endemic areas are characterised by high temperatures and low rainfall (50–1000 mm per year), often with a short period of heavy rainfall. Actinomycetoma is more commonly observed in areas with higher rainfall and lower temperatures, whilst eumycetoma is found in areas with lower rainfall and less variation in temperature (Bakshi and Mathur, 2008; Hassan et al., 2021). Young immunocompetent adults living in rural areas are predominantly affected. Countries reporting the highest number of cases are Sudan (*n* = 10,608), Mexico (*n* = 4,155) and India (*n* = 1,116) (Emery and Denning, 2020) although epidemiological surveillance for mycetoma is absent in most endemic countries and the true burden of mycetoma globally is not known (van de Sande, 2013; World Health Organization, 2018).

### 1.1 Eumycetoma causative agents

Eumycetoma is caused by more than 40 species of fungi, all of which belong to the phylum Ascomycota (Ahmed et al., 2019). The causative agents of eumycetoma either produce dark grains, which are dark-brown to black in colour, or pale grains which are white to yellow. The most common cause of eumycetoma globally is *Madurella mycetomatis* (order Sordariales), which produces black grains (van de Sande, 2013). Other organisms that commonly cause dark grain mycetoma belong to the order Pleosporales and include *Falciformispora senegalensis*, *Trematosphaeria grisea*, *Nigrograna mackinnonii* and *Medicopsis romeroi* (van de Sande, 2013; Ahmed et al., 2018). The most common causative agents of pale grain eumycetoma belong to the *Scedosporium apiospermum* species complex; others include *Acremonium* spp., *Fusarium* spp. and *Aspergillus* spp. The causative agents of eumycetoma are summarised in Table 1, adapted from Ahmed et al. (2019) “Fungi causing eumycotic mycetoma”. (Ahmed et al., 2019).

**TABLE 1:** Eumycetoma causative agents by grain colour.

	Dark grain (brown-black)	Pale grain (white-yellow)
Order	Species	Species
Sordariales	*Madurella mycetomatis*	
	*Madurella pseudomycetomatis*	
	*Madurella tropicana*	
	*Madurella fahalii*	
Pleosporales	*Falciformispora senegalensis (syn. Leptosphaeria senegalensis)*	*Neotestudina rosatii*
	*Falciformispora tompkinsii*	
	*Trematosphaeria grisea (syn. Madurella grisea)*	
	*Medicopsis romeroi (syn. Pyrenochaeta romeroi)*	
	*Nigrograna mackinnonii (syn. Pyrenochaeta mackinnonii)*	
	*Curvularia lunata*	
	*Curvularia geniculata*	
	*Pseudochaetosphaeronema larense*	
	*Corynespora cassiicola*	
	*Emmarelia grisea*	
	*Emmarelia paragrisea*	
Microascales		*Scedosporium apiospermum complex*
		*Microascus gracilis*
		
Chaetothyriales	*Exophiala jeanselmei*	
	*Cladophialophora* spp*.*	
Diaporthales	*Phialophora verrucosa*	*Phaeoacremonium krajdenii*
		*Phaeoacremonium parasiticum*
Hypocreales		*Fusarium solani*
		*Fusarium oxysporum*
		*Fusarium falciforme*
		*Fusarium verticillioides*
		*Sarocladium kiliense*
		*Acremonium recifei*
		*Ilyonectria destructans (syn. Cylindrocarpon destructans)*
		*Phialophora cyanescens (syn. Cylindrocarpon cyanescens)*
Eurotiales		*Aspergillus flavus*
		*Aspergillus fumigatus*
		*Aspergillus nidulans*
Calosphaeriales		*Pleurostomophora ochracea*
Onygenales		*Microsporum* spp*.*
	= common causative agents	

### 1.2 Treatment of eumycetoma

The treatment of eumycetoma is unsatisfactory. Treatment involves prolonged antifungal therapy in combination with surgery, and decisions regarding choice of antifungal agent are based on drug availability and limited data from observational studies. Itraconazole is the most commonly used antifungal agent, and terbinafine is a frequently used alternative; however the cure rate using these antifungals in combination with surgery is 25%–30% (Zein et al., 2012; Sow et al., 2020). In many cases terbinafine is used after failure with itraconazole, although it has been used in combination with itraconazole (Estrada et al., 2012; Sow et al., 2020). Recurrence occurs in almost one-third of cases (Wadal et al., 2016). Voriconazole or posaconazole may be preferred in certain situations although experience with these agents is limited, and use of liposomal amphotericin B in a few cases has been disappointing (Estrada et al., 2012; Mattioni et al., 2013). Due to the chronicity of the disease process, most cases develop a high degree of fibrosis, which prevents the antifungals from reaching the tissue adequately. Often patients present late with advanced infection, which entails a worse outcome.

There is an urgent need for more effective antifungal treatments. This review will provide an update on the latest developments in the treatment of eumycetoma, including data on currently available antifungals and candidate drugs which could provide therapeutic benefit.

## 2 Antifungal susceptibility testing for eumycetoma causative agents

*In vitro* susceptibility assays have been developed for many of the causative agents of black grain eumycetoma. These are based on the M38–A3 Clinical and Laboratory Standards Institute (CLSI) broth microdilution reference method for moulds and use viability dyes such as resazurin and tetrazolium salts to detect metabolic activity (Ahmed et al., 2004; Abd Algaffar et al., 2021; van de Sande, 2021). *M. mycetomatis* is the most studied of the causative agents; it has a low 50% minimum inhibitory concentration (MIC50) for the azoles (median MIC50 0.03 μg/mL) and higher MICs for amphotericin B (MIC50 0.5 μg/mL) and terbinafine (MIC50 8 μg/mL) (van de Sande, 2021). Similar susceptibility patterns are seen with *F. senegalensis*, *Trematospheria grisea* and *N. mackinnonii*, which show relatively low MICs for itraconazole (MIC50 0.125–0.5 μg/mL), posaconazole (MIC50 0.03–0.125 μg/mL) and voriconazole (MIC50 0.25–0.5 μg/mL), and higher MICs for amphotericin B (MIC50 0.5–8 μg/mL). *Madurella mycetomatis* is not inhibited by 5-flucytosine (MIC50 > 64 μg/mL) or the echinocandins (MIC50 of 64 μg/mL for caspofungin, and >128 μg/mL for both anidulafungin and micafungin) (Ahmed et al., 2015a; Abd Algaffar et al., 2021). Azole and allylamine combinations (ketoconazole with terbinafine, and itraconazole with terbinafine) *in vitro* have not shown any synergistic effect against *M. mycetomatis* (Ahmed et al., 2015b). Antifungal susceptibility testing is not routinely performed in clinical settings where mycetoma is endemic, therefore limited data are available to correlate with clinical outcomes.

## 3 Evaluating drug efficacy—*in vivo* models

**FIGURE 2:**
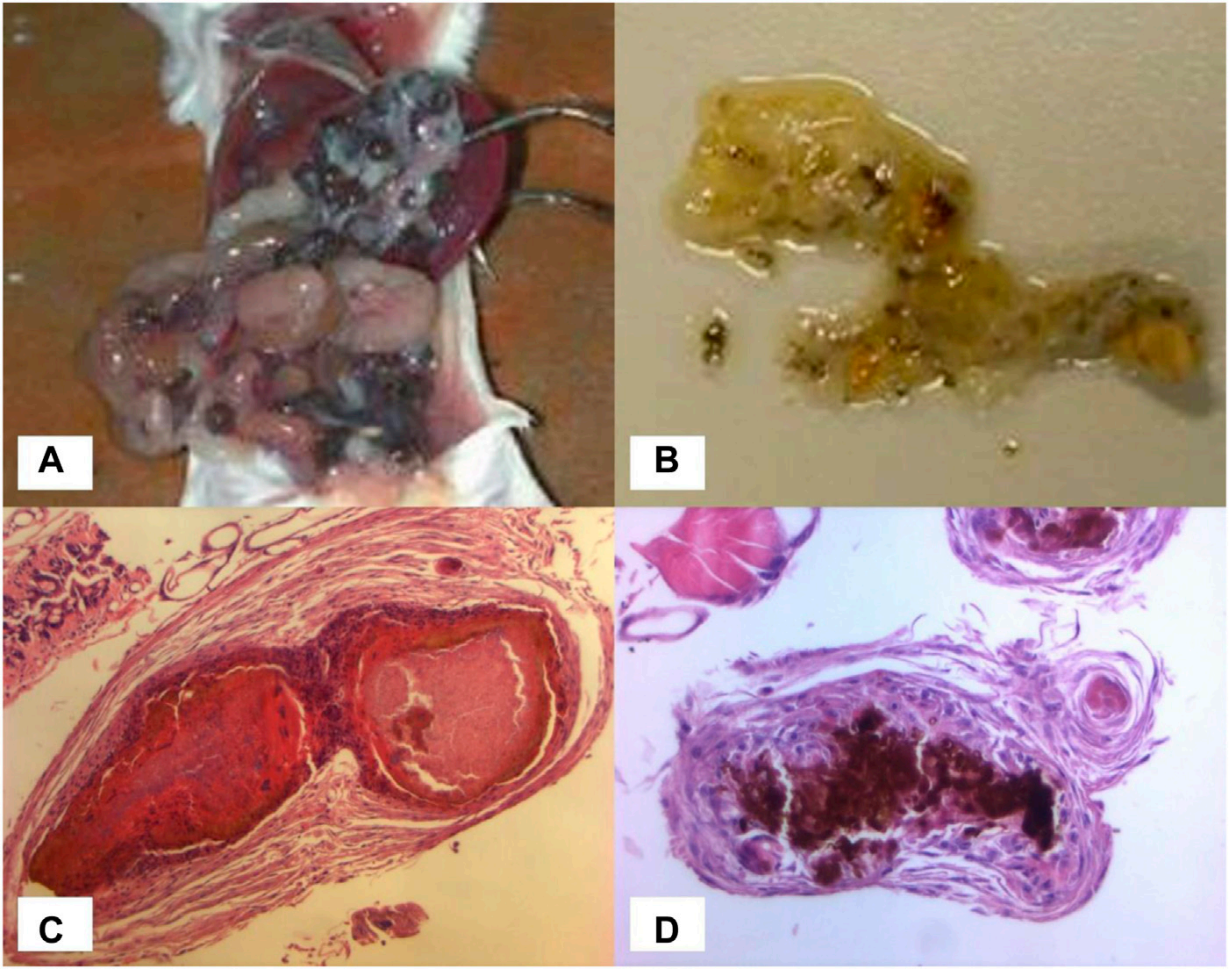
*In vivo* models. **(A)** Numerous black grains within the abdominal cavity of a BALB/c mouse. **(B)** Black grains within a dissected *Galleria mellonella* larva. **(C)** Grain formation in *Galleria mellonella* larva at day 3, histological specimen (H&E x10). **(D)** Grain formation in *Galleria mellonella* larva at day 3, histological specimen (H&E x20).

Limited work has been conducted using animal models. Murine intraperitoneal grain models have been described, in which BALB/c mice are infected by intraperitoneal injection of *M. mycetomatis* mycelium plus sterile soil as an adjuvant (MURRAY et al., 1960; Ahmed et al., 2003). These mice develop numerous black grains within the abdominal cavity (Figure 2A) with histological features comparable to those seen in human infection. The earliest study conducted by Murray in 1962 (MURRAY and COLICHON, 1962) assessed the efficacy of diamidinodiphenylamine in a murine intraperitoneal grain model, but failed to show any reduction in the number of grains in treated *versus* non-treated mice.

All subsequent studies have been published since 2015 using murine and invertebrate models of *M. mycetomatis* infection. The invertebrate model is a *G. mellonella* (greater wax moth) grain model of *M. mycetomatis* infection (Kloezen et al., 2015). Larvae of *Galleria mellonella* are inoculated by injecting a suspension of viable *M. mycetomatis* into the last left pro-leg, and subsequently develop grains resembling those seen in both human and murine infection (Figures 2B–D). Grain formation in larvae occurs as early as 4 h after inoculation, and by day 3 the presence of cement material within grains, and a collagen-like capsule surrounding grains, is observed. The immune response in *G. mellonella* larvae involves both a humoral response, and a cellular response which is mediated by hemocytes (phagocytic cells similar to neutrophils). The cellular immune reaction in *G. mellonella* is observed as early as 4 h after exposure to *M. mycetomatis* hyphae but seems most intense at day 7 when the collagen capsule disappears. In murine models, a large neutrophil zone is observed surrounding encapsulated grains. Using a BALB/c murine intraperitoneal grain model (Ahmed et al., 2003) itraconazole was shown to be ineffective against a strain of *M. mycetomatis* that had been highly susceptible to itraconazole *in vitro* (MIC 0.06 μg/mL); whereas treatment of these mice with amphotericin B prevented grain formation despite the lower susceptibility of that strain to amphotericin B *in vitro* (MIC 0.5 μg/mL) (van de Sande et al., 2015). These findings were replicated in the *G. mellonella* grain model of *M. mycetomatis* infection, in which survival was unaltered in *G. mellonella* larvae treated with azoles (ketoconazole, itraconazole and voriconazole), but was enhanced in larvae treated with amphotericin B or terbinafine (Kloezen et al., 2018). The most likely explanation is that azole antifungals are ineffective against *M. mycetomatis in vivo* due to their inability to penetrate grains; a hypothesis which was confirmed by enhanced efficacy of itraconazole when melanin, one of the *M. mycetomatis* grain constituents, was inhibited. For posaconazole (van de Sande et al., 2015) enhanced survival was also noted in larvae treated with a higher dose (14 mg/kg *versus* 5.7 mg/kg posaconazole) demonstrating that the drug concentration is also important.

The lack of synergistic effect observed from combining azoles with terbinafine *in vitro* (Ahmed et al., 2015b) has also been demonstrated *in vivo*. No difference in survival was observed in *G. mellonella* larvae treated with itraconazole and terbinafine together, compared with larvae treated with either agent alone (Eadie et al., 2017). Additionally, antagonism has been demonstrated in larvae treated with a combination of amphotericin B plus itraconazole or terbinafine, compared with larvae treated with amphotericin B alone (Eadie et al., 2017).

## 4 Novel antifungal agents and approaches to treatment

*In vitro* susceptibility assays for the eumycetoma causative agents have been used to screen large numbers of molecules for their potential therapeutic effect; some of these molecules have been screened because they are active against other fungal pathogens, others as part of the open source drug discovery project for mycetoma named MycetOS. Initially these efforts focused on *M. mycetomatis*, as the commonest cause of eumycetoma, although more recently the screening of these compounds has been widened to assess their activity against other causative agents.

Of the antifungal agents currently in use for other fungal infections, to date amphotericin B, the azoles ketoconazole, miconazole, itraconazole, fluconazole, voriconazole, posaconazole, isavuconazole, ravuconazole, luliconazole, lanoconazole and otesoconazole, the allylamine terbinafine, the echinocandins caspofungin, anidulafungin and micafungin, the orotomide olorofim, and 5-flucytosine have been evaluated (van de Sande et al., 2005; van Belkum et al., 2011; Ahmed et al., 2014; Ahmed et al., 2015b; Lim et al., 2018; van de Sande, 2021; Lim et al., 2022a; Nyuykonge et al., 2022a; Nyuykonge et al., 2022b).

Of the antifungal agents screened for activity against *M. mycetomatis*, the lowest MICs obtained were for ravuconazole (MIC50 0.008 mg/L) and olorofim (MIC50 0.016 mg/L) (Ahmed et al., 2014; Lim et al., 2020; Nyuykonge et al., 2022b). Both of these agents have been shown to enhance survival in *G. mellonella* larvae infected with *M. mycetomatis*. (Lim et al., 2022a). Higher MICs were obtained for amphotericin B and terbinafine, and for the echinocandins and 5-flucytosine no activity was noted.

Ravuconazole has a broad spectrum of activity, demonstrating *in vitro* activity against the parasite *Trypanosoma cruzi* (Urbina et al., 2003) and a range of fungal pathogens including *Exophiala jeanselmei*, *Curvularia lunata*, and *Aspergillus* spp. (Fung-Tomc et al., 1998; Cuenca-Estrella et al., 2005) In phase 1/2 trials both ravuconazole and its prodrug E1224 (fosravuconazole l-lysine ethanolate) demonstrated favourable pharmacokinetic properties and were well tolerated (Groll et al., 2005). Fosravuconazole is available in intravenous and oral formulations, and following oral administration achieves serum concentrations much higher than the MIC90 for *M. mycetomatis* (Ahmed et al., 2014). High tissue concentrations are achieved following oral administration, including in skin, nail and deeper tissues such as adipose and bone marrow (Groll et al., 2005). A clinical trial of fosravuconazole *versus* itraconazole for the treatment of eumycetoma caused by *M. mycetomatis* in Sudan is nearing completion. Clinical breakpoints are not available for any of the antifungal agents, although recently epidemiological cut-off values (ECVs) have been proposed for ravuconazole and itraconazole. Using 131 clinical isolates of *M. mycetomatis*, MICs ranged from 0.002 to 0.125 mg/L for ravuconazole, compared with 0.008–1 mg/L for itraconazole (Nyuykonge et al., 2022b). The ECV for ravuconazole was set at 0.064 mg/L and the ECV for itraconazole at 1 mg/L.

Olorofim is a member of the novel orotomide class of antifungals that target the enzyme dihydroorotate dehydrogenase (DHODH) leading to inhibition of the pyrimidine biosynthesis pathway. Olorofim has demonstrated activity against an extended range of fungal pathogens including azole-resistant *Aspergillus* species (Buil et al., 2017). In earlier studies *Aspergillus fumigatus* and *M. mycetomatis* were shown to have 58.7% homology in DHODH sequences and to share 6 out of 7 amino acids involved in the predicted binding mode of olorofim, indicating that *M. mycetomatis* was likely to be susceptible to olorofim (Oliver et al., 2016; Lim et al., 2020). MICs for olorofim ranged from 0.004 to 0.125 mg/L.

At this time approximately 2,100 compounds have been screened for their activity against *M. mycetomatis in vitro*; 1,600 originating from the Medicines for Malaria Venture (MMV) open boxes, and 510 donated or generated by the MycetOS community itself (Lim et al., 2018; Lim et al., 2022a; Lim et al., 2022b; OpenSourceMycetoma, 2023). From those 1,600 compounds screened, 404 inhibited *M. mycetomatis* growth at a concentration of 100 μM and 26 had an IC50 < 8 µM. Of these 23 were tested *in vivo,* and 9 demonstrated *in vivo* efficacy. The nine compounds with *in vivo* efficacy included the azoles bitertanol, posaconazole and ravuconazole, the orotomide olorofim, the aminothiazole MMV006357, the antifolate MMV675968, the benzimidazoles fenbendazole and MMV1782387, and MMV022478. Based on the lead compounds identified from these screenings, most emphasis in the drug screening efforts were on six different compound series, namely, the fenarimols (series 1), aminothiazoles (series 2), phenothiazines (series 3), antifolates (series 4), benzimidazoles (series 5) and the ketoximes (series 6). The first series focused around the fenarimol analogue EPL-BS1246. Initially 35 out of an 800 fenarimol compound library were screened for *in vitro* activity, and the five active compounds were also tested *in vivo.* Using the *G. mellonella* grain model, three of these compounds (EPL-BS0178, EPL-BS0495 and EPL-BS1025) significantly prolonged larval survival; the other two compounds (EPL-BS1246 and EPL-BS0800) did not prolong larval survival, however a significant reduction in the number and size of grains was observed (Lim et al., 2018).

When the chemical properties of the compounds able to prolong larval survival were compared to those that were not, a correlation with lipophilicity in terms of LogD was noted (Lim et al., 2018). In additional work, more molecules were produced and tested and it was confirmed that fenarimol analogues with a low LogD were indeed more likely to penetrate the mycetoma grain and enhance larval survival. However, the fenarimol analogues, like the azoles are inhibitors of sterol 14alpha-demythelases (CYP51) which are essential for sterol biosynthesis in eukaryotes. This means that they are not the most suitable candidates for combination therapy. Therefore in other compound series, compounds with a different mode of action are investigated.

A combination which appeared more successful was combining melanin inhibitors with itraconazole. Melanin production is a recognised virulence factor of several fungal pathogens that cause superficial and subcutaneous infection. Grains formed by *M. mycetomatis* in tissue consist of tightly packed fungal hyphae within an amorphous brown cement-like material. The constituents of this cement material include melanin along with heavy metals, proteins and lipids, which are thought to have a protective effect against antifungal agents (Ibrahim et al., 2013). The causative agents of dark grain eumycetoma synthesise melanin via 1,8-dihydroxynaphthalene (DHN)-, 3,4-dihydroxyphenylalanine (DOPA)- and pyo-melanin biosynthetic pathways. *Madurella mycetomatis* produces melanin by DHN- and pyo-melanin pathways, and this has been shown to protect against the action of itraconazole and ketoconazole *in vitro*, increasing MICs 16-fold and 32-fold, respectively (van de Sande et al., 2007). *M. romeroi* and *F. senegalensis* synthesise melanin via DHN-, DOPA- and pyo-melanin pathways, whilst *Trematosphaeria grisea* and *Falciformispora tompkinsii* only produce DHN-melanin (Lim et al., 2021). Treatment of *M. mycetomatis*-infected *G. mellonella* larvae with DHN- and DOPA-melanin inhibitors results in the production of non-melanised grains, and treatment with itraconazole in combination with the DHN-melanin inhibitors carpropamid and fenoxanil significantly enhanced larval survival, compared to treatment with itraconazole alone (Lim et al., 2022c). Carpropamid and fenoxanil act on the enzyme scytalone dehydratase within the DHN melanin biosynthesis pathway to inhibit the production of 1,3,8–trihydroxynaphthalene reductase. Glyphosphate inhibits DOPA-melanin synthesis by acting on the enzyme 5-enolpyruvoylshikimate 3-phosphatesynthase (EPSPS) in the shikimate pathway. Interestingly, treatment with itraconazole in combination with either carpropamid or glyphosphate resulted in increased grain melanisation compared to treatment with either carpropamid or glyphosphate alone, suggesting that under conditions of stress due to treatment with itraconazole *M. mycetomatis* is able to upregulate melanin synthesis via alternative pathways. The observed increase in survival in larvae treated with itraconazole and carpropamid is not fully understood, however the failure to observe an increase in survival in larvae treated with itraconazole and glyphosphate suggests that this effect is independent of grain melanisation. These findings suggest a potential role for melanin inhibition as a therapeutic adjunct in cases of eumycetoma caused by *M. mycetomatis*, however further work is needed to understand the complex pathways that regulate melanin biosynthesis and to evaluate the effectiveness of this approach against other eumycetoma causative agents.

A limited number of antifungal agents demonstrate a broader spectrum of antifungal activity, inhibiting the Pleosporalean fungi (*F. senegalensis*, *M. romeroi* and *T. grisea*) in addition to *Madurella* species including *M. mycetomatis*, *M. pseudomycetomatis*, *M. tropicana* and *M. fahalii*. The most active of these are luliconazole and lanoconazole (median MICs 0.001–0.064 μg/mL for both) and ravuconazole (median MICs 0.008–2 μg/mL) (Nyuykonge et al., 2022a). Other compounds capable of inhibiting the growth of *Madurella* spp. (*M. mycetomatis*, *M. pseudomycetomatis*, *M. tropicana*), *F. senegalensis* and *M. romeroi* include fenbendazole and carbendazim (benzimidazole carbamates), tafenoquine (8-aminoquinolone derivative) and MMV1578570 (Lim et al., 2022a).

In addition to looking at chemical compound libraries, also natural compounds have been evaluated. Natural products including plant extracts and essential oils are used frequently in traditional herbal remedies in endemic settings. Some of these plants, such as *Melaleuca alternifolia, Boswellia papyrifera*, *Acacia nubica* and *Nigella sativa*, are known to have antifungal properties and have been shown to inhibit the growth of *M. mycetomatis in vitro* (Elfadil et al., 2015). CIN-102 is a synthetic cinnamon oil blend (trans-cinnamaldehyde 86.7% w/w) which has demonstrated activity against *M. mycetomatis in vitro*, with MICs ranging from <32 μg/mL to 512 μg/mL (Konings et al., 2021). In the *G. mellonella* larval model, the highest concentration of CIN-102 administered (800 mg/kg) was toxic, however at concentrations below the MIC (40 mg/kg and 80 mg/kg), larval survival was significantly enhanced. In the larvae demonstrating enhanced survival, there was no reduction in the number or size of grains, however earlier encapsulation of the grains and large hemocytes (invertebrate immune cells) were observed suggesting an immunomodulatory effect of CIN-102. Further work is needed to better understand the mechanism of action of CIN-102 (Table 2).

**TABLE 2:** New and emerging antifungal agents for eumycetoma: pre-clinical data.

Class of drug	*In vitro* susceptibility		*In vivo* efficacy (*M. mycetomatis*)	Comments
	*M. mycetomatis*, MIC50 or range (ug/mL unless stated otherwise)	Pleosporalean fungi*, IC range		
Triazoles				
Mechanism of action: inhibition of lanosterol demethylase in the ergosterol biosynthesis pathway				
Itraconazole	0.03	0.125–8	No - not in invertebrate model or mouse model	
Posaconazole	<0.03	0.03–1	Yes - improved survival in invertebrate model (dose-dependent)	
Voriconazole	0.06	0.125–1	No - not in invertebrate model	
Isavuconazole	0.03	NA	NA	
Ravuconazole	0.008	0.008–8	Yes - improved survival in invertebrate model	Fosravuconazole in clinical trial
Bitertanol	0.06 uM	NA	Yes - improved survival in invertebrate model	In use as a pesticide
Difenoconazole	0.06 uM	NA	Yes - reduced number and size of grains	In use as a pesticide
Imidazoles				
Mechanism of action: inhibition of lanosterol demethylase in the ergosterol biosynthesis pathway				
Ketoconazole	0.125	NA	No - not in invertebrate model	
Luliconazole	0.001	0.004–0.064	NA	Only available in topical formulation
Lanoconazole	0.002	0.008–0.125	NA	Only available in topical formulation
Tetrazoles				
Mechanism of action: inhibition of lanosterol demethylase in the ergosterol biosynthesis pathway				
Oteseconazole	1 uM	NA	No - not in invertebrate model	
Allylamines				
Mechanism of action: inhibition of squalene epoxidase in the ergosterol biosynthesis pathway				
Terbinafine	8	NA	Yes - improved survival in invertebrate model	
Echinocandins				
Mechanism of action: inhibition of 1,3-beta-D-glucan synthase in the cell wall biosynthesis pathway				
Caspofungin	64	8 - >16	NA	
Anidulafungin	128	NA	NA	
Micafungin	128	NA	NA	
Orotomides				
Mechanism of action: inhibition of dihydroorotate dehydrogenase in the de novo pyrimidine biosynthesis pathway				
Olorofim	0.016	NA	Yes - improved survival in invertebrate model	
Polyenes				
Mechanism of action: binds directly to ergosterol in the cell membrane				
Amphotericin B	0.5	0.125–16	Yes - improved survival in invertebrate model, prevented grain formation in mouse model	
Fenarimol analogues				
Mechanism of action: putative inhibition of lanosterol demethylase in the ergosterol biosynthesis pathway				
EPL-BS1246	1	NA	Yes - reduced number and size of grains	
EPL-BS0178	8	NA	Yes - improved survival in invertebrate model	
EPL-BS0495	4	NA	Yes - improved survival in invertebrate model	
EPL-BS1025	4	NA	Yes - improved survival in invertebrate model	
Salicylanilides				
Mechanism of action: induces mitochondria-to-nucleus retrograde response				
Niclosamide	0.78–1.6	NA	NA	
Niclosamide-ethanolamine	0.78–1.6	NA	NA	
MMV665807	1.6	NA	NA	
Benzimidazole carbamates				
Mechanism of action: binds to β-tubulin and destabilises microtubule formation				
Fenbendazole	0.5	Inhibits growth	Yes - improved survival in invertebrate model	
Carbendazim	0.5	Inhibits growth	Yes - partially improved survival in invertebrate model	
MMV1782387	4	Inhibits growth	Yes - improved survival in invertebrate model	
8-Aminoquinolines				
Mechanism of action: unknown				
Tafenoquine	4	Inhibits growth	No - not in invertebrate model	
Strobilurins				
Mechanism of action: binds to cytochrome b to inhibit mitochondrial respiration				
Azoxystrobin	0.06	NA	Yes - reduced number and size of grains	In use as a pesticide
Trifloxystrobin	0.25	NA	Yes - reduced number and size of grains	In use as a pesticide
Aminothiazole derivatives				
Mechanism of action: inhibition of calcium-activated small conductance potassium channels				
MMV006357	0.25	NA	Yes - improved survival in invertebrate model	
Folate inhibitors				
Mechanism of action: dihydrofolate reductase (DHFR) inhibition				
MMV675968	2	NA	Yes - partially improved survival in invertebrate model	
Pyrazolopyrimidines				
Mechanism of action: unknown				
MMV022478	4	NA	Yes - partially improved survival in invertebrate model	
Quinoline derivatives				
Mechanism of action: unknown				
Iodoquinol	8	NA	NA	
Plant extracts and essential oils				
Mechanism of action: unknown				
CIN-102	128	NA	Yes - improved survival in invertebrate model	
Other				
Mechanism of action: unknown				
MMV1578570	Inhibits growth	Inhibits growth	NA	

*includes Falciformispora senegalensis, Trematosphaeria grisea, Nigrograna mackinnonii and Medicopsis romeroi.

NA—data not available

## 5 Future developments and challenges

Mycetoma is recognised by the WHO as a Neglected Tropical Disease, and the causative agents of eumycetoma are identified as ‘high priority’ pathogens in the recently published WHO Fungal Priority Pathogens List (World Health Organization, 2022). Fungal diseases are known to receive less than 2% of the funding allocated to infectious diseases research in the United Kingdom and internationally (Head et al., 2014; World Health Organization, 2022). Additionally, of the 19 fungal pathogens included in the WHO priority list, eumycetoma causative agents ranked second for unmet research and development need.

Of the available antifungal agents, fosravuconazole is promising for use in the near future. It is the first treatment for eumycetoma to have been evaluated in a clinical trial, and has the advantage over other antifungal agents of once weekly administration which is likely to help with compliance.

It is important to highlight that the search for an ideal antifungal lies not only in its direct action against etiological agents, with low MIC, but also in the ability to achieve adequate tissue concentrations in chronic diseases such as mycetoma and chromoblastomycosis where significant fibrosis limits the penetration of antifungal agents into infected tissue. Novel formulations of antifungal agents could be explored as a means of enhancing tissue bioavailability and drug efficacy. Various nanomedicine strategies and nano-drug delivery systems have been used to enhance drug penetration and uptake in models of tumours in which there is a highly fibrotic stroma (Lu and Prakash, 2021). The use of a self-nanoemulsifying delivery system (SNEDDS) was shown to enhance the efficacy of ravuconazole in *T. cruzi*-infected mice (Spósito et al., 2021).

Actinomycetoma has a high cure rate when treated with combination antibiotic therapy, however our understanding of safe and efficacious combinations of antifungal treatments for eumycetoma is extremely limited. Currently there is no effective fungicidal agent. The combination of olorofim with an azole requires further investigation; however olorofim, like the azoles, is fungistatic. The best approach might involve two or more antifungal agents (including an effective fungicidal agent) in combination with a melanin inhibitor, in a formulation that allows better delivery and penetration into infected tissue. This strategy would be worth exploring for other fungal NTDs, especially chromoblastomycosis.

Recent *in vivo* studies have used the *G. mellonella* grain model of *M. mycetomatis* infection to evaluate drug efficacy. The most promising drugs should also be evaluated in mammalian models to better understand their efficacy and toxicity, however there is need to develop better animal models. The murine intraperitoneal model does form typical grains however the anatomical location of the grains is not representative of human infection. Past attempts to develop subcutaneous models of infection, including in monkey paw and mouse footpad, have failed and this should be addressed (MURRAY et al., 1960; Cavanagh, 1974). Future work should also develop *in vivo* models of infection with other eumycetoma causative agents. Recently a *G. mellonella* grain model of *F. senegalensis* infection has been developed (unpublished).

Data on the efficacy of existing antifungal treatments are needed to guide management decisions and inform treatment guidelines. There is also a need to develop more effective antifungal agents and adjunctive therapies, and to identify synergistic antifungal combinations. Future studies need to correlate clinical outcomes with microbiological data including the results of *in vitro* susceptibility testing, ideally using molecular tools to identify the causative organism. In order to achieve this, it is essential to strengthen laboratory capacity in endemic settings and ensure sustainable investment in research and development.
